# Selenium, Selenoprotein Genes and Crohn’s Disease in a Case-Control Population from Auckland, New Zealand

**DOI:** 10.3390/nu4091247

**Published:** 2012-09-07

**Authors:** Liljana Gentschew, Karen S. Bishop, Dug Yeo Han, Angharad R. Morgan, Alan G. Fraser, Wen Jiun Lam, Nishi Karunasinghe, Bobbi Campbell, Lynnette R. Ferguson

**Affiliations:** 1 Discipline of Nutrition, FM&HS, University of Auckland, Private Bag 92019, Auckland, 1142, New Zealand; Email: l.gentschew@ikmb.uni-kiel.de (L.G.); dy.han@auckland.ac.nz (D.Y.H.); w.lam@auckland.ac.nz (W.J.L.); b.campbell@auckland.ac.nz (B.C.); 2 Auckland Cancer Society Research Center, FM&HS, University of Auckland, Private Bag 92019, Auckland, 1142, New Zealand; Email: k.bishop@auckland.ac.nz (K.S.B.); n.karunasinghe@auckland.ac.nz (N.K.); 3 Nutrigenomics New Zealand, University of Auckland, Private Bag 92019, Auckland, 1142, New Zealand; Email: angharad_r_morgan@hotmail.com (A.R.M.); a.fraser@auckland.ac.nz (A.G.F.); 4 Department of Medicine, University of Auckland, Private Bag 92019, Auckland, 1142, New Zealand

**Keywords:** selenium, Crohn’s Disease, risk factor, single nucleotide polymorphism

## Abstract

New Zealand has one of the highest incidence rates of Crohn’s Disease (CD), whilst the serum selenium status of New Zealanders is amongst the lowest in the world. A prospective case-control study in Auckland, New Zealand considered serum selenium as a potential CD risk factor. Serum selenium levels were significantly lower in CD patients compared to controls (101.8 ± 1.02 *vs.* 111.1 ± 1.01 ng/mL) (*p *= 5.91 × 10^−8^). Recent detailed studies in the United Kingdom have suggested an optimal serum level around 122 ng/mL, making the average CD patient in New Zealand selenium deficient. Of the 29 single nucleotide polymorphisms (SNPs) tested, 13 were found to significantly interact with serum selenium on CD. After adjustment for multiple testing, a significant interaction with serum selenium on CD was found for three SNPs, namely rs17529609 and rs7901303 in the gene *SEPHS1*, and rs1553153 in the gene *SEPSECS*. These three SNPs have not been reported elsewhere as being significantly associated with selenium or CD. It is unclear as to whether lower selenium levels are a cause or an effect of the disease.

## 1. Introduction

Inflammatory bowel disease (IBD) represents a chronic idiopathic intestinal inflammatory condition that causes significant changes in neuronal controlled gut functions, resulting in dysfunction of the intestinal tract and a higher mortality rate than for the general population [1]. On the basis of clinical, radiological, and histological criteria, IBD has been traditionally categorized as ulcerative colitis (UC) and Crohn’s Disease (CD). CD, rather than UC, appears to have a multifactorial character, in which a genetic predisposition, the external environment, intestinal microbial flora, and the immune system are all involved in disease susceptibility [2,3,4]. 

Low selenium levels have been found amongst people with IBD [5,6], and low levels have also been linked to a number of other health problems including: decreased survival in people infected with HIV [7], epileptic seizures in children and adults [8] as well as a number of age-associated neurological disorders [9]. New Zealand has one of the highest incidence rates of IBD in the world and more research is necessary to identify further risk factors for addressing public health needs. The selenium status of children and adults in parts of New Zealand is amongst the lowest (an intake of 31–38 µg/day dependent on age group) in the world [10,11] and there is an urgent need to reduce the risk of possible selenium deficiency. Due to the low selenium status and high incidence rates of CD in New Zealand [12], the relationship between selenium status and CD is of interest.

Unlike most other nutrients, the intake of selenium varies enormously worldwide [13]. Seafood, poultry, and eggs are the main source of selenium in New Zealand [14], although grains and various crops can be a good source depending on the selenium content of the soil in which they were grown. Selenium intake in New Zealand has increased due to a higher importation of Australian wheat and cereal products and an increased selenium supplementation in animal feed in New Zealand [15]. However, selenium status in New Zealand is still considered to be marginal and may not be adequate for the full activity and protective effects of selenoproteins [11,16]. Due to the wide variability in the selenium content of different foods; the huge range of selenium intake from one geographical region to another; and lifestyle factors such as smoking and intense exercise which may increase selenium requirements, the consequence for health needs to be established on an individual basis. In addition, numerous factors such as selenium source, plasma selenium level, intake of other nutrients (such as Vitamins C and E) and the individual ability to synthesize selenoproteins, determine the effects of selenium status on an individual [13,17,18]. 

The aim of the present study was to investigate the effect of serum selenium level in modulating the risk of CD and to evaluate the interaction with specific single nucleotide polymorphisms (SNPs) in genes coding for selenoproteins or genes indirectly involved in selenoprotein synthesis. Ultimately the intention was to draw conclusions that could be applied to personalized nutrition in clinical practice.

## 2. Experimental Section

A prospective case-control, population-based study was performed by the Nutrigenomics New Zealand programme to explore the association of serum selenium levels with CD. Study subjects were recruited in New Zealand between May 2005 and November 2010 (by the Discipline of Nutrition and the Department of Medicine the Faculty of Medical and Health Sciences, University of Auckland). Clinical records were analysed to confirm IBD status and phenotypic status was assigned according to the Montreal Classification System [19,20] providing a useful framework for genotype-phenotype analysis. To reduce ethnic variability only those who self-reported Caucasian ancestry were included [4]. Due to very small numbers it was not possible to analyse data from the other ethnic groups. The study was conducted under ethical protocol MEC/04/12/011, authorised through the New Zealand Multi-Region Human Ethics Committee. All study subjects gave informed consent. A total of 1204 (351 CD patients and 853 controls) participants were available for serum selenium association with CD, and up to 306 CD patients and 492 controls were available for genotyping and had serum selenium levels available. 

Demographic information was collected by means of a validated questionnaire and selenium was measured in serum samples by Gribbles Laboratory, Hamilton, New Zealand. Genotyping was performed on DNA isolated from peripheral blood samples or buccal swabs as described by Morgan *et al.* [21]. 

Published articles and a number of databases (Hap Map, UCSC Genome Browser and NCBI) were used to select SNPs within the following genes: *DIO1*, *DIO2*, *GPX1*, *GPX3*, *SEPHS1*, *SEPSECS* and *TXNRD2*. These genes were chosen as the Wellcome Trust Case Control Consortium observed an association between CD and SNPs in some of these genes, and hence we suspected there may be a serum selenium-gene interaction. A multiple correction was applied to the gene-serum selenium interaction on CD data by calculating *q*-values (based on *p*-values) in order to apply the false discovery rates method [22]. 

Haploview Software (Haploview 4.2) was used to select SNPs by choosing tagged SNPs, as well as data on linkage disequilibrium and haplotype block analysis. The target genomic region was extended 5 kb upstream and downstream of the genes. A minor allele frequency of ≥0.03 in the Caucasian population was selected in order to avoid the use of rare SNPs as our sample size would be too small to be informative. If two SNPs were highly correlated (*r*^2^ ≥ 0.8), only one SNP was selected. In total, 29 SNPs in 7 genes were selected and samples were genotyped using the Sequenom^®^ Mass ARRAY technology and iPlex system as outlined in Morgan *et al.* [23].

Across group comparisons of serum selenium concentration were tested using a generalized linear model for mean differences. The serum selenium levels in the study group were not normally distributed and therefore were log-transformed. The estimates made from the analyses were then back-transformed using an exponential (anti-log) function to be consistent with the original measurements/units. A *p*-value of less than 0.05 was considered significant. These analyses were carried out using R (R: A language and environment for statistical computing, R Foundation for Statistical Computing, Vienna, Austria. ISBN 3-900051-07-0) [24] and SAS (Version 9.2, SAS Institute, Cary, NC, USA) [25].

## 3. Results

**Figure 1:**
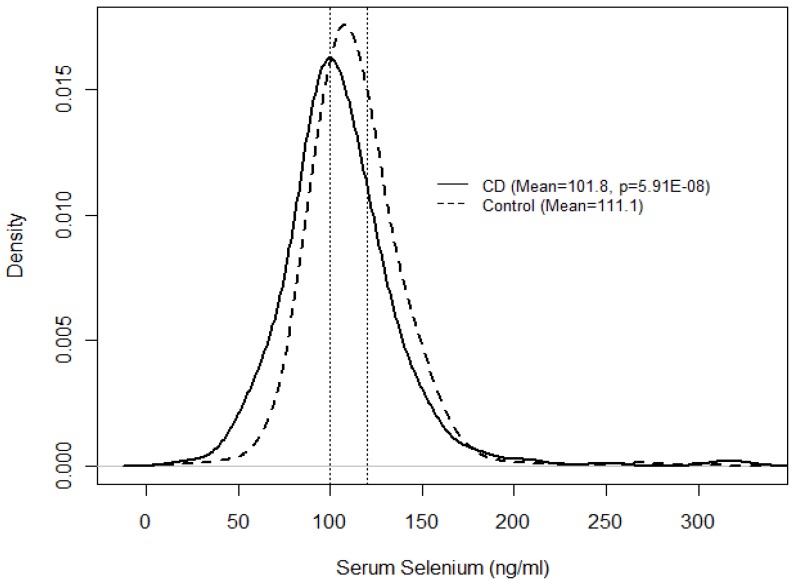
Distribution of serum selenium measures by CD and control group.

Serum selenium levels were significantly lower in CD patients compared to controls (*p *= 5.91 × 10^−8^, Table 1). The mean serum selenium concentration for CD patients was 101.8 ng/mL and 111.1 ng/mL for the controls (Table 1 and Figure 1). Serum selenium was divided into tertiles representing low (<100.5 ng/mL), medium (100.5–118.5 ng/mL) and high levels (>118.5 ng/mL). Thereby, a significantly higher risk of having CD was observed in individuals in the lower selenium group as compared with those in the high selenium group and compared with those in the medium selenium group. No significant difference was found between medium and high selenium groups (Table 2), suggesting that there is a level of serum selenium that is adequate and higher levels may offer no benefit.

**Table 1:** Odds ratio of Crohn’s Disease (CD) status with serum selenium.

	*N* (%)	Serum selenium (ng/mL)		
		Mean (SE)	OR (95% CI)	*p*
CD	351 (29.2)	101.8 (1.02)	0.25 (0.15–0.42)	5.91 × 10^−8^
Control	853 (70.8)	111.1 (1.01)	1.00	

**Table 2:** Odds ratio of Crohn’s Disease (CD) status with grouped serum selenium levels.

Grouped serum selenium (ng/mL)	CD *N* (%)	Control *N* (%)	OR (95% CI)	*p*	OR (95% CI)	*p*
Low (<100.5)	170 (48.4)	234 (27.5)	2.35(1.73–3.20)	**5.10 × 10^−12^**	2.61(1.92–3.55)	**8.67 × 10^−10^**
Medium (100.5–118.5)	89 (25.4)	320 (37.6)	0.9(0.65–1.25)	0.996	1.00	
High (>118.5)	92 (26.2)	298 (35.0)	1.00			

There was no significant interaction of serum selenium and smoking status on CD (*p* = 0.237). However, ever-smokers showed a higher risk of having CD in low and medium selenium groups than non-smokers (OR = 2.23, *p* = 0.0012; and OR = 1.94, *p* = 0.0161, respectively) when association between CD status and smoking status within grouped selenium level was tested.

Study participants, who were diagnosed with CD between the ages of 17 and 40 years of age, comprised 71.5% of the cohort. CD affected the colon, the ileum and both ileum and colon in CD patients as shown in Table 3. The following classifications were reported in the CD study participants: inflammatory, penetrating and stricturing CD.

**Table 3:** Frequency and mean serum selenium level with estimate by CD and sub-phenotypes.

	*N* (%) *	Mean serum selenium (ng/mL) (SE)	Estimate (95% CI)	*p*
**Age at first diagnosis**				
0–16 years	43 (12.9)	91.2 (1.04)	0.821 (0.765–0.881)	6.57 × 10^−8^
17–40 years	239 (71.5)	103.4 (1.02)	0.931 (0.899–0.965)	7.56 × 10^−5^
Over 40 years	52 (15.6)	104.5 (1.05)	0.941 (0.881–1.006)	0.073
**CD location**				
Ileal	122 (36.5)	101.2 (1.03)	0.911 (0.870–0.954)	7.80 × 10^−5^
Colonic	108 (32.3)	107.9 (1.03)	0.971 (0.927–1.018)	0.226
Ileocolonic	104 (31.2)	97.0 (1.03)	0.873 (0.832–0.916)	4.10 × 10^−8^
**CD Behaviour**				
Inflammatory	182 (54.7)	103.5 (1.02)	0.932 (0.897–0.969)	3.22 × 10^−4^
Stricturing	110 (33.0)	97.5 (1.04)	0.878 (0.836–0.923)	2.85 × 10^−7^
Penetrating	41 (12.3)	106.8 (1.03)	0.962 (0.895–1.033)	0.285
**CD location/Behaviour**				
Colonic/Inflammatory	78 (60.5)	109.3 (1.03)	0.984 (0.932–1.039)	0.559
Ileal/Stricturing	51 (39.5)	103.5 (1.06)	0.932 (0.870–0.998)	0.043
Any relative with IBD (Yes)	101 (30.2)	105.1 (1.03)	0.947 (0.902–0.993)	0.026
Bowel resection (Yes)	119 (35.5)	99.4 (1.03)	0.895 (0.854–0.938)	3.49 × 10^−6^
Any EIMs (Yes)	63 (18.8)	106.6 (1.04)	0.960 (0.905–1.019)	0.182
Perianal disease (Yes)	48 (14.4)	100.3 (1.04)	0.903 (0.844–0.966)	0.003
**Smoker at diagnosis (CD) or recruitment (Controls) **				
CD	106 (45.1)	100.0 (1.03)	0.905 (0.850–0.958)	6.54 × 10^−4^
Control	224 (29.3)	110.5 (1.02)	1.00	

* data was not available for all participants; SE: Standard error; IBD: inflammatory bowel disease; EIM: extraintestinal manifestations; CI: Confidence interval.

Serum selenium level was also compared between sub-phenotypes and controls (Table 3). Serum selenium level was significantly lower when CD was diagnosed before the age of 40 years and no significant association was found amongst those diagnosed when over 40 years. Serum selenium level was significantly lower when CD was located in the ilium and ileocolon rather than in the colon alone. Likewise, when CD was classified as inflammatory or stricturing, rather than penetrating, the serum selenium levels were significantly lower. CD patients who had bowel resection or perianal disease had significantly lower serum selenium levels. Smoking status was tested with serum selenium levels as well as CD status. Serum selenium levels were significantly lower in CD patients who smoked compared with controls who smoked (*p* = 6.54 × 10^−4^). In addition, there was a significant difference between CD and controls with respect to smoking status (*p* = 7.22 × 10^−6^).

Gene-serum selenium interaction analysis with CD was performed on 29 SNPs located in seven genes (Table 4). Except for *SEPHS1* and *SEPSECS* all other genes studied code for selenoproteins. 

**Table 4:** List of SNPs tested in this study.

Gene	Name	Chr	SNP	Tested allele
*DIO1*	deiodinase, iodothyronine, type I	1	rs12095080	A
			rs2294511	A
			rs2294512	A
			rs731828	A
*DIO2*	deiodinase, iodothyronine, type II	14	rs10136454	C
			rs12885300	C
			rs225011	C
			rs225012	G
			rs225014	C
			rs1800668	C
*GPX1*	glutathione peroxidase 1	3	rs3763015	A
*GPX3*	glutathione perosidase 3 (plasma)	5	rs3792796	C
			rs3792797	A
			rs3828599	A
			rs8177412	C
			rs8177425	C
			rs870407	A
			rs10752294	C
*SEPHS1*	selenophosphate synthetase 1	10	rs11258337	A
			rs17529609	G
			rs7901303	T
			rs11937742	A
*SEPSECS*	Sep ( *O*-phosphoserine) tRNA:Sec (selenocysteine) tRNA synthase	4	rs13123725	A
			rs1553153	A
			rs17480524	C
			rs2302565	C
			rs7666342	A
			rs1548357	T
*TXNRD2*	thioredoxin reductase 2	22	rs5748469	A

Thirteen SNPs: the variant rs1800668 of the *GPX1*, rs3792797 of the *GPX3*, three SNPs (rs1295080, rs2294511, and rs2294512) of the *DIO1*, two SNPs (rs225011 and rs225012) of the *DIO2*, two SNPs (rs17529609 and rs7901303) of the *SEPHS1*, three SNPs (rs11937742, rs1553153, and rs2302565) of the *SEPSECS*, and rs1548357 of the *TXNRD2* genes significantly interacted with serum selenium (Table 5). After correcting for multiple testing two SNPs in *SEPHS1* and one SNP in *SEPSECS* (rs1553153) remained significant (Table 5). No other SNPs in the genes listed in Table 5 remained significantly associated with gene-serum selenium interaction with CD after multiple corrections was applied.

**Table 5:** Gene-serum selenium interaction on CD.

Interaction serum selenium with Gene		Tested allele	CD	
Gene	SNP		Estimate (SE)	*p*
*GPX1*	rs1800668	C	−1.685 (0.762)	0.0270
*GPX3*	rs3792797	A	−1.526 (0.755)	0.0431
*DIO1*	rs12095080	A	−1.902 (0.852)	0.0255
*DIO1*	rs2294511	A	−0.942 (0.480)	0.0497
*DIO1*	rs2294512	A	−1.005 (0.486)	0.0388
*DIO2*	rs225011	C	−1.242 (0.555)	0.0253
*DIO2*	rs225012	G	−1.179 (0.552)	0.0327
*SEPHS1*	rs17529609	G	−1.740 (0.617)	**0.0048** *
*SEPHS1*	rs7901303	T	−1.117 (0.420)	**0.0078** *
*SEPSECS*	rs11937742	A	−1.000 (0.435)	0.0214
*SEPSECS*	rs1553153	A	−1.754 (0.623)	**0.0048** *
*SEPSECS*	rs2302565	C	−0.891 (0.428)	0.0374
*TXNRD2*	rs1548357	T	−0.792 (0.403)	0.0490

* remain significant after a multiple correction applied using false discovery rate.

## 4. Discussion

In a Caucasian population residing in New Zealand we evaluated 29 SNPs in seven genes that might play a direct or indirect role in CD pathogenesis. Some of the SNPs associated with CD in this cohort have been reported elsewhere [23,26,27,28], whilst others do not appear to have been reported. Here we focus on the gene-serum selenium interaction on CD. 

Low serum selenium was found to be significantly associated with CD. No significant difference was found between medium and high serum selenium cases and controls (Table 1 and Table 2), suggesting that there is a level of serum selenium that is adequate. There have been previous reports on low selenium status in CD patients, especially in young patients [29,30]. Levels as low as those found amongst nearly half of the CD patients in this study have been shown to have negative implications for the general maintenance of genomic stability [31]. Selenium is known to be depleted in CD patients on enteral nutrition [32,33], but this was not the form of nutritional intake for most of this group. Thus, an important question that is unanswered by this study is whether this association is a cause or an effect of the disease.

The failure of some large selenium human intervention studies to improve human health, e.g., the SELECT trial [34], has raised questions as to what serum levels of selenium should be attained for optimal human health, and whether supplementation is beneficial. A recent review by Rayman suggests 122 ng/mL is desirable, and levels both below and above this threshold may be undesirable [35]. On the current analysis, most of the control subjects had levels close to this optimal level, whereas many of the CD patients were significantly deficient in this mineral. It may be appropriate to consider supplementation for such individuals.

With respect to smoking, in those with a low or medium serum selenium level, there was a significant association with CD. It appears that high serum selenium levels may be protective against smoking-related risks of CD and the therapeutic effects amongst smokers with CD could be important to explore further. Cigarette smoking is known to have a detrimental effect with respect to the risk of CD and course of disease [36,37]. Surprisingly, smoking is a protective factor in patients with UC [38].

In the present study, SNPs in prioritized genes have been statistically analyzed to investigate whether, at a given serum selenium level, the SNPs were associated with a different risk of CD. Interestingly, one SNP in *GPX1* (rs1800668), one SNP in *GPX3* (rs3792797), three SNPs in *DIO1* (rs12095080, rs2294511 and rs2294512), two SNPs in *DIO2* (rs225011 and rs225012), two SNPs in *SEPHS1* (rs17529609 and rs7901303), three SNPs in *SEPSECS* (rs11937742, rs1553153 and rs2302565) and one SNP in *TXNRD2* (rs1548357) (Table 5) were significantly associated with modified selenium levels indicating that these genes might increase the risk of developing CD.

In IBD, oxidative stress plays a major role in disease pathogenesis. GPX 1–4 and 6 are selenium dependent [39] and some of the most important antioxidant enzymes in humans and therefore it is possible that SNPs in *GPX1* may be involved in modulating several diseases. GPX1 is a ubiquitous enzyme with antioxidant properties, and plays an important role in antioxidative defense [40,41] and therefore a dysfunction of *GPX1* might be involved in CD pathogenesis. In this study we found that rs1800668 (C/T) in *GPX1* is significantly associated with a gene-serum selenium interaction on CD (*p* > 0.027) but did not remain significant after adjustment for multiple testing. The rs1800668 polymorphism has not been widely studied or found to be significantly associated with disease. One of the difficulties associated with GPX activity is that it can change over time depending on lifestyle factors, and vary from one cell type to another [42]. Hansen *et al*. found that only 5% of the total variation in GPX (in erythrocytes) could be accounted for by genotype and lifestyle factors such as smoking, alcohol and fruit and vegetable intake [42]. 

Interestingly, previous studies found that altered expression and activity of DIOs is present in several models of heart failure, brain injury, tumor tissues and local inflammation [43,44,45,46]. These findings support the hypothesis that SNPs in DIOs may play an important role in the development of CD. However, in this study none of the ten SNPs tested in the *DIO1* and *DIO2* genes were found to significantly interact with gene-serum selenium with CD after multiple corrections was applied. The role of selenium in modifying thyroid hormone levels is controversial [47,48,49]. Patients with autoimmune thyroiditis and a baseline serum selenium levels around 83 ng/mL supplemented with 200 µg/day selenomethionine for 3 or 6 months have shown no changes in free thyroxin or free tri iodothyronine levels [50], suggesting that DIO activity is saturated at these serum selenium levels. The current study group has a serum selenium level of 101 ng/mL which is higher than the levels recorded in Anastasilakis *et al.* [50]. Therefore, it is unlikely that selenium level has a direct influence on *DIO1* activity or thyroid function in this group. 

The effect of selenium on the thyroid hormone metabolism might be due to lower cytokine release that results in a higher activity of *DIO1*. Therefore, selenium deficiency might lower the *DIO1* activity by lower availability of selenium for *DIO1* synthesis. It is unlikely that selenium supplementation has a direct influence on *DIO1* activity or thyroid function, but selenium indirectly improves clinical conditions which lead to normalization of thyroid functions. Furthermore, the effect of selenium depends on the tissue. For example, the thyroid and pituitary are more resistant to dietary selenium compared to the liver [51]. In addition, interaction effects between selenium and other dietary nutrients such as iodine exists, and some of these interactions that modify thyroid hormone metabolisms and antioxidant status have been described elsewhere [52,53,54]. It was not possible to consider these other dietary nutrients in this study.

SNPs in both *SEPSECS* and *SEPHS1* were found to significantly interact with serum selenium levels and CD, even after correction for multiple testing. The three SNPs that have been shown to be significant have not been reported elsewhere. *SEPHS2* has been shown to be essential for Selenocysteine synthesis, but this is not so for *SEPHS1* [55]. It is possible that *SEPHS1* is involved in the regulation of oxidative stress and cell growth [56]. The interaction of *SEPHS1* with p53 has been reported by Chung *et al*. [57]. Gender specific p53 variants have been associated with CD [58]. The p53 mutations in UC related cancers have also been shown [59]. This implies a possible interaction of *SEPHS1* with the mutated p53 pathway causing a malabsorption of selenium. The gut inflammation in IBD could be affecting the function of p53 [60] and causing downstream influence on other pathways such as the functionality of *SEPHS1*.

It is possible that the *SEPSECS* is involved in CD development due to their involvement in the synthesis of selenocysteine. However, the functional consequences or secondary effects are more likely to be directly related to CD pathogenesis. Genetic variants in *SEPSECS* may lead to insufficient function resulting in an inappropriate synthesis of selenoproteins which in turn lead to decreased selenium levels. However, the exact function of *SEPSECS* and *SEPHS1* is still unclear and further studies are needed to investigate their role in CD development. Further studies are required to link genetic variants in selenoproteins and associated entities with modified selenium levels and altered selenium metabolism. 

## 5. Conclusions

In summary, low serum selenium was found to be significantly associated with CD in a Caucasian population in New Zealand. A significant association was found between smoking and CD amongst those with a low or medium serum selenium level. In order to investigate whether specific genotypes were associated with CD in terms of modifying individual selenium levels, SNPs in prioritized genes were analyzed. Three SNPs in *SEPSECS* and *SEPHS1* were found to significantly interact with serum selenium level and CD, even after correction for multiple testing. The exact function of *SEPSECS* and *SEPHS1* remains unclear and further studies are necessary to investigate their role in CD development, as well as the potential protective effect of selenium in those who smoke. Further studies are required to link genetic variants in selenoproteins and related entities with modified selenium levels and altered selenium metabolism that could bring about functional changes that may be implicated in the pathogenesis of CD.
